# Recurrent dizziness due to paroxysmal atrioventricular block following carotid artery stenting in a patient with a normal atrioventricular conduction and iatrogenic carotid sinus syndrome

**DOI:** 10.1016/j.hrcr.2024.06.007

**Published:** 2024-06-19

**Authors:** Joud Fahed, Rayna Isber, Nidal Isber

**Affiliations:** ∗ Department of Cardiology, Richmond University Medical Center, Staten Island, New York; † Barnard College, Columbia University, New York, New York

**Keywords:** Carotid artery stenting, Carotid sinus reflex, Bradycardia, Hypotension, Paroxysmal atrioventricular block, Carotid hypersensitivity syndrome

**Key Teaching Points**

• Carotid artery stenting (CAS) is commonly associated with sinus bradycardia in the periprocedural period and up to 1 week postprocedure.
• Our case demonstrated that bradycardia following CAS can also be due to the ominous paroxysmal atrioventricular block and may persist for months.
• We hypothesized that carotid artery stent might have produced a condition similar to carotid sinus syndrome owing to the mechanical stretch of the baroreceptors in the carotid sinus causing enhanced vagal tone.

## Introduction

Patients who suffer from carotid artery disease are at high risk for ischemic stroke, especially in those with high-grade stenosis of 80% or more. Carotid artery stenting (CAS) is one of the therapeutic options that gained popularity in recent years for the treatment of carotid stenosis. CAS is a catheter-based procedure, performed via the transfemoral approach under local anesthesia and without heavy sedation. Hence, in many cases, it is usually preferred over the traditional, more invasive surgical option of carotid endarterectomy.

However, in the periprocedural period, CAS can be associated with hemodynamic instability resulting from the development of bradycardia and hypotension. Stretching of the carotid sinus baroreceptors by the balloon and the stent is believed to cause carotid sinus reflex, leading to these manifestations. Hypotension and bradycardia usually occur at the time of balloon inflation and settle down immediately or within 24 hours of the procedure. The risk of bradycardia and hypotension is influenced by the proximity of the stenosis to the carotid bulb and the magnitude of the dilation performed. Treatment of CAS-related hemodynamic instability includes administration of vasopressors like dopamine and phenylephrine, and some persistent cases may require permanent pacemaker (PPM) implantation.

We report a patient with CAS who experienced recurrent dizziness for 3 months following the procedure and was found to have episodes of paroxysmal atrioventricular block (PAVB) on a cardiac monitor. He was treated with a dual-chamber permanent pacemaker (PPM), which resulted in the resolution of his symptoms. Rapid atrial pacing both during His bundle study at the time of pacemaker implantation and during follow-ups showed normal atrioventricular (AV) conduction. According to our knowledge and upon reviewing the medical literature, there have been no previous reports of bradycardia or PAVB developing after CAS later than 10 days after the procedure.

## Case report

The patient is an asymptomatic 76-year-old man who was referred for the evaluation of left carotid artery stenosis found on a routine screening test with Doppler ultrasound. His past medical history includes hypertension, hypercholesterolemia, hypothyroidism, gout, and peripheral arterial disease status post stent placement in the right leg. The patient denies any history of stroke, transient ischemic attack, lightheadedness, dizziness, syncope, or bradycardia. Further testing with magnetic resonance imaging revealed evidence of an ulcerated, irregular plaque at the left carotid bifurcation and proximal portion of the left internal carotid artery, resulting in approximately 85% stenosis using the NASCET criteria. Therapy with angioplasty and stent placement was recommended. The patient underwent intervention on September 14, 2023, using a 6 × 8 × 40 mm stent that was deployed across the stenosis. Two balloons (3 mm × 20 mm, 5 mm × 20 mm) were used to perform angioplasty pre- and post-procedure, respectively.

**Figure 1:**
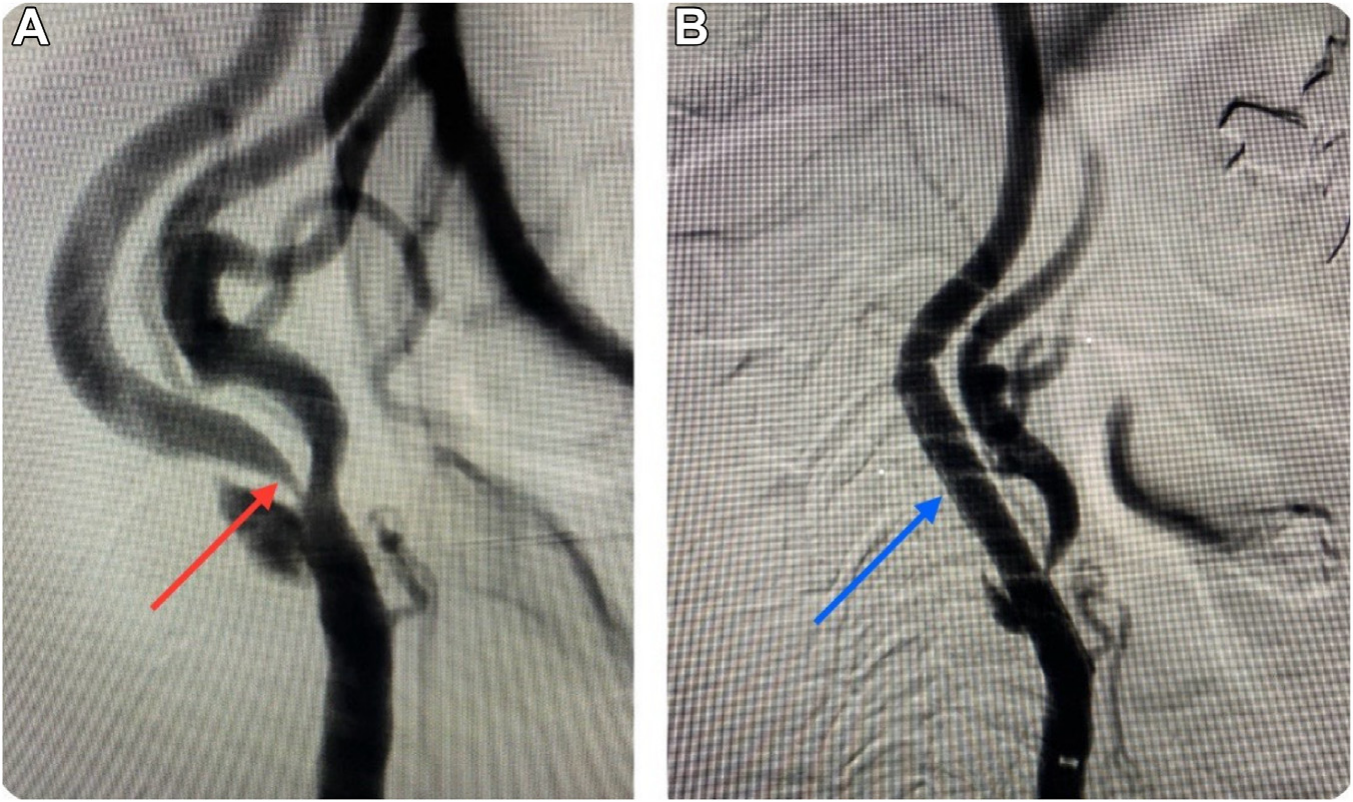
Digital subtraction angiogram of the carotid artery stenosis before and after carotid artery stenting (CAS). **A:** Image shows almost 85% stenosis of the left internal carotid artery at the level of the carotid sinus (*red arrow*). **B:** The same image projection after placement of CAS (*blue arrow*). The internal carotid arterial narrowing was fully relieved by angioplasty and stenting.

**Figure 2:**
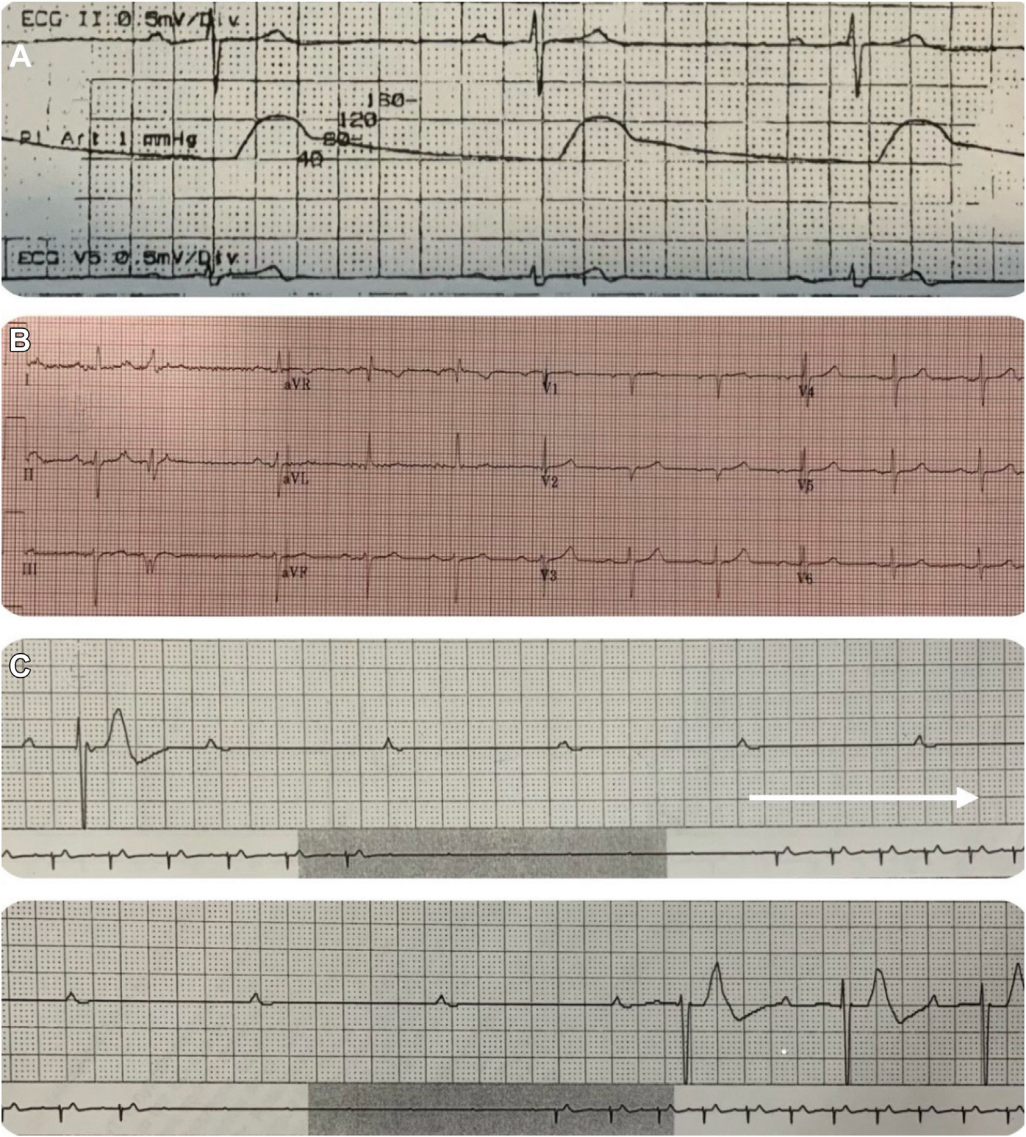
**A:** Severe sinus bradycardia during the procedure. **B:** Twelve-lead electrocardiogram prior to pacemaker implantation. **C:** Holter tracing shows pauses owing to vagally mediated paroxysmal atrioventricular (AV) block for 9.3 seconds. AV block starts suddenly and ends suddenly without premature atrial contractions or premature ventricular contractions. There is slowing of the sinus rate (P-P interval) before and during the pause and a delay of AV conduction (prolonging PR).

Immediately after stent placement (Figure 1), the patient developed hemodynamic instability with a drop-in heart rate to 40–50 beats per minute (bpm) from a baseline heart rate in the 70s (Figure 2A) and a decline in blood pressure (BP) to 80/50 mm Hg from a baseline BP around 130/80 mm Hg. He was treated with atropine, phenylephrine, and intravenous fluids and was admitted for observation in the intensive care unit. The patient continued to have bradycardia and hypotension for several days, requiring repeated treatment. On the fifth day, the patient was found to be stable and was discharged home.

Three months after CAS, the patient presented to his primary care physician a week after an episode of presyncope. He complained of intense dizziness such that he had to hold on to the wall to prevent himself from falling. The patient also had recurrent episodes of lightheadedness at rest and while exercising, but he ignored them. He denied any loss of consciousness, palpitations, shortness of breath, or chest pain. His physical examination, including BP and heart rate, was within normal limits. His 12-lead electrocardiogram showed normal sinus rhythm, mild PR prolongation (224 ms), and left axis deviation (Figure 2B).

**Figure 3:**
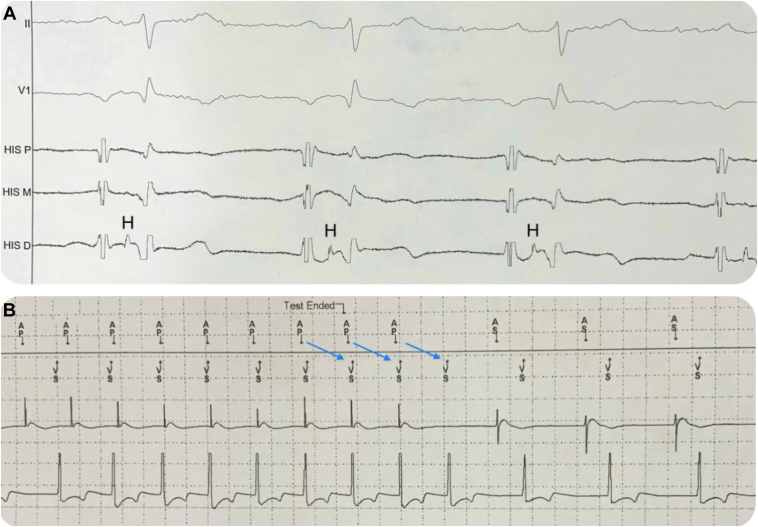
**A:** Tracing shows His bundle study, which demonstrates normal HV interval of 55 ms. **B:** Tracing shows atrial pacing at 140 beats per minute, which resulted in 1:1 atrioventricular (AV) conduction, confirming normal AV conduction system.

A 24-hour Holter monitor was ordered, which revealed 50 episodes of bradycardia longer than 2 seconds. These episodes were caused by a high degree of paroxysmal AV nodal block, with the longest one at about 9.3 seconds (Figure 2C). The patient was immediately admitted to the hospital, and on December 12, 2023, he had a PPM implanted. His bundle study at the time of pacemaker implantation revealed normal AV nodal and infranodal conduction. The AH interval was 140 ms and the HV interval was 55 ms (Figure 3A). Atrial pacing at 140 bpm resulted in 1:1 AV conduction. Furthermore, laboratory tests showed adenosine serum levels 10.27 U/L, within normal range (0.00–15.00 U/L).

On 1-week and 2-month follow-ups postimplant, atrial pacing showed 1:1 AV conduction at 140 bpm (Figure 3B), indicating a normal AV conduction system. These findings suggest that the PAVB that the patient experienced is due to enhanced vagal tone caused by stimulation of the carotid sinus’s baroreceptors by the stent, leading to this patient’s symptoms.

## Discussion

In this case report, we describe a patient with a new onset of recurrent dizziness due to PAVB for 3 months after having CAS. Electrophysiology study showed normal AV node and infrahisian conduction. Therefore, it is likely that the cause of bradycardia is an increase in parasympathetic activity. Carotid stenting seemed to have produced carotid hypersensitivity–like syndrome. Implantation of PPM resulted in a complete resolution of the patient’s symptoms. To our knowledge, there have been no previous reports of PAVB occurring for 3 months after CAS.

The carotid sinus is located in the adventitia of the carotid bulb of the internal carotid artery. It contains baroreceptors (stretch receptors) that are sensitive to changes in pressure and play a crucial role in maintaining hemodynamic stability through their effect on BP and heart rate. Through Hering’s nerve, a branch of the glossopharyngeal nerve (the ninth cranial nerve), afferent signals from the carotid sinus are transferred to the midbrain. Meanwhile, efferent signals travel from the midbrain via parasympathetic nerves in the vagus and sympathetic nerves to the heart and blood vessels.^1^

According to the literature, the rate of bradycardia and hypotension after CAS varies from 27% to 37% and from 10% to 42%, respectively.^2^^,^^3^ Several studies have identified various risk factors for this phenomenon, including the presence of the calcification,^4^ eccentric plaque, proximity to the carotid bifurcation,^5^ type of the stent selected, balloon-to-artery diameter ratio, concurrent contralateral lesions, positive stress test, history of coronary artery disease, urgent versus selected procedures,^6^, ^7^, ^8^ and asymptomatic and elderly patients.^5^ Most patients who undergo CAS are monitored for delayed occurrence of bradycardia and hypotension for a minimum of 12 hours postprocedure.^2^ However, some authors reported the occurrence of sinus bradycardia 1 week after the procedure.^9^ To our knowledge, there have been no previous reports of PAVB or very delayed occurrence of any time of bradycardia after CAS.

PAVB, defined by Rosenbaum and colleagues,^10^ is an abrupt, complete AV block in a patient with otherwise 1:1 conduction, with delayed ventricular escape leading to a period of asystole followed by resumption of AV conduction. There are 3 subtypes of PAVB,^11^ including intrinsic phase 4, vagally mediated, and idiopathic. It can cause dizziness and syncope, depending on the duration of asystole. Intrinsic PAVB is usually a pause-dependent phase 4 that occurs in the diseased His-Purkinje conduction system. Meanwhile, the idiopathic subtype occurs in patients with low baseline levels of adenosine, rendering them hypersensitive to adenosine surges resulting in AV block. However, our patient’s electrophysiology study showed a normal AV conduction system, and measurements of plasma adenosine levels showed values within normal ranges.^12^ Even though adenosine plasma level measurement is not a routinely performed test, and there are no specific tests for diagnosis of idiopathic AVB since it is a diagnosis of exclusion, we obtained measurements to further help rule out the possibility of the idiopathic AVB. Moreover, the rhythm strip of 1 of the episodes of PAVB demonstrated a gradual increase in P-P cycle lengths and PR prolongation and a gradual decrease in sinus rate (Figure 2C).^13^ These findings lead us to presume that his symptoms are due to vagally mediated PAVB. This subtype occurs secondary to a surge in vagal parasympathetic and inhibition in sympathetic activity, resulting in carotid hypersensitivity–like syndrome. In patients with carotid hypersensitivity syndrome, turning or shaving the neck or looking upward may produce bradycardia and may cause dizziness or syncope. In addition, carotid stimulation by the stent, as in our case, is one of its common triggers. Bradycardia caused by carotid hypersensitivity syndrome can manifest with sinus bradycardia, AV block, or both.

The prevalence of PAVB is not known, and it is considered an ominous arrhythmia owing to its propensity to syncope and sudden cardiac death. Therefore, prompt recognition is essential because sudden cardiac death may be preventable by PPMs. Pacemaker implantation has high efficacy in intrinsic and idiopathic PAVB, with less than 5% recurrence of syncope. Meanwhile, there is a higher incidence rate of recurrence (5%–20%) in vagally mediated PAVB.^14^ Even though pacemaker implantation is the mainstay treatment, some authors reported successful treatment with cardioneuroablation.^15^ This procedure targets ganglionated plexuses to denervate the AV node, aiming to eliminate the cardioinhibitory reflex. However, most studies are single center, and further large-scale studies are required to establish the clinical utility, efficacy, and safety of this treatment for vagally mediated PAVB.^15^

## Conclusion

We presented a patient with a new onset of recurrent dizziness due to PAVB developing late after CAS was treated successfully with PPM. Since the patient had normal AV conduction on electrophysiology study, the most plausible etiology for the bradycardia is enhanced vagal tone owing to intermittent stimulation of the carotid sinus by the stent. We hypothesize that movement of the neck in specific directions may be the trigger for carotid sinus stimulation by the stent, and that stenting may produce a condition that may be similar to carotid sinus syndrome. This is the first report implicating a possible connection between carotid stenting and carotid hypersensitivity syndrome.

Our case is important because it demonstrates that bradycardia can be due to PAVB and persists for longer periods, which prompts us to continue monitoring the patients and taking into consideration each symptom that the patient may report.

## Disclosures

The authors declare no conflict of interest regarding this article.
